# Pharmacological Interventions in Older Adults

**DOI:** 10.1093/geroni/igaf122.1647

**Published:** 2025-12-31

## Abstract

This session describes opportunities for pharmacological management in older adults as well as deprescribing in older adults.

